# Use of echocardiographic pulmonary acceleration time and estimated vascular resistance for the evaluation of possible pulmonary hypertension

**DOI:** 10.1186/1476-7120-11-7

**Published:** 2013-02-27

**Authors:** Sven-Olof Granstam, Erik Björklund, Gerhard Wikström, Magnus W Roos

**Affiliations:** 1 Department of Medical Sciences Clinical Physiology, Uppsala University, Uppsala, Sweden; 2 Department of Medical SciencesCardiology, Uppsala University, Uppsala, Sweden

**Keywords:** Echocardiography, Right heart catheterization, Acceleration time, Systolic pulmonary pressure, Pulmonary vascular resistance, Pulmonary hypertension

## Abstract

**Background:**

During ultrasound examination, tricuspid regurgitation may be absent or gives a signal that is not reliable for the estimation of systolic pulmonary pressure. The aim of this study was to evaluate the usefulness of acceleration time (AT) from the right ventricular outflow tract (RVOT) as an estimation of the trans-tricuspid valve gradient (TTVG) and to investigate the correlation between estimated and invasive pulmonary vascular resistance (PVR).

**Methods:**

The AT was correlated to the TTVG measured with routine standard echocardiography in 121 patients. In a subgroup of 29 patients, systolic pulmonary pressure (SPAP) and mean pulmonary arterial pressure (MPAP) were obtained from recent right heart catheterization (RHC).

**Results:**

We found no significant correlation between the estimation of right atrial pressure (RAP) by echocardiography and the RAP obtained by RHC. Estimated SPAP (TTGV + RAP mean from RHC) showed a good linear relation to invasively measured SPAP. TTVG and AT showed a non-linear relation, similar to SPAP and MPAP measured by catheterization and AT. For detection of SPAP above 38 mmHg a cut-off for AT of 100 ms resulted in a sensitivity of 89% and a specificity of 84%. For detection of MPAP above 25 mmHg a cut-off for AT of 100 ms resulted in similar sensitivity and specificity. Invasive PVR and the ratio of TTVG and the time velocity integral of the RVOT (TVI _RVOT_ ) had a strong linear relation.

**Conclusions:**

Our study confirms that AT appears to be useful for the evaluation of pulmonary hypertension. In high risk patients, an AT of less than 100 ms indicates a high probability of pulmonary hypertension. Furthermore, PVR estimation by ultrasound seems preferably be done by using the ratio of TTVG and TVI _RVOT_.

## Background

Right ventricular (RV) pressure is routinely estimated by analysing the tricuspid regurgitation (TR) Doppler signal. This is especially important when screening for pulmonary hypertension (PH) in high risk patients for example with systemic sclerosis (SSc) [1] or HIV [2]. Often the TR is absent or gives a signal that is not reliable [3]. In such cases information from a possible pulmonary insufficiency can be used [4,5]. Further, measurement of the pulmonary flow acceleration time (AT) could be used for the estimation of RV pressure and systolic pulmonary pressure (SPAP) [6]. The aim of the study was to evaluate the usefulness of the AT based on using noninvasive and invasive data. Moreover, calculating pulmonary vascular resistance (PVR) using RHC is of great importance when evaluating patients with suspected or known PH. As previously suggested by Abbas *et al.,* a formula for noninvasive estimation of PVR [7] was used. However, recently this formula has been questioned in the subset of more severe PH including high PVR [8]. In the present study with high prevalence of PH the RHC-derived PVR was therefore compared with the PVR estimated by using the formula of Abbas *et al*. Furthermore, alternative ways of estimating echocardiographic PVR either with heart rate in the formula as suggested by Haddad *et al.*[9], or without were evaluated.

## Methods

Between June 2010 and December 2011, echocardiographic data that could be further analysed for both AT and TTVG was obtained from 121 patients at Uppsala University Hospital and retrospectively analysed. To be included in the study the Doppler signals had to be of adequate quality for measurements. RHC was performed in 29 of these patients within two days from echocardiography and data from the two methods were compared in this subgroup. In Uppsala University Hospital the Department of Cardiology is a referral centre for pulmonary arterial hypertension (PAH) which provides the echocardiographic laboratory with a large group of patients with suspected pulmonary hypertension (PH) defined as SPAP above 38 mmHg. This level of SPAP corresponds to mean pulmonary arterial pressure (MPAP) of above 25 mmHg. This level of estimated SPAP has been suggested for further investigation for PH [10], because these patients are likely to develop PH. Both patients with and without PH were included.

### Echocardiography

Ultrasound examination was performed mainly using Philip^,^s IE 33 (Bothel, WA), and in a minority General Electric^,^s Vivid e9 (GE Healthcare, Little Chalfont, UK). Patients were retrospectively included when the quality of both TTVG and AT was satisfactory for evaluation. Systolic right ventricular (RV) pressure was estimated from the velocity of the TR. The forward velocity profile, obtained by pulsed Doppler in the RVOT close to the pulmonary valve, was used to obtain AT and TVI _RVOT_. The AT was measured in accordance with previous studies [6,9,11], defined as the time from the onset to maximal velocity. The sweep speed was approximately 75 mm/s and obtained values are the average of 3 beats. In addition the information from the pulmonary velocity profile and TR were used to evaluate a previously suggested formula [7], PVR = TR max velocity/ TVI _RVOT_ · 10 + 0.16. Alternative ways of estimating PVR from TTVG and TVI _RVOT_ were evaluated, either with heart rate taken into account (TTVG/ (TVI _RVOT_ · heart rate)) or not (TTVG/ TVI _RVOT_) [8,9]. Noninvasive estimations of RAP were based on size and collapse of the ICV in these studies [9,10]. In our study it was decided before the study started that only use RAP by cava estimation if this correlated well to invasively measured RAP in the RHC subgroup. If the RAP values were not well correlated the mean RAP obtained in the RHC subgroup would be used. Furthermore, in the whole group correlation with AT to TTGV instead of estimated SPAP would be performed.

### Right heart catheterization

In a subgroup of 29 patients, both RHC data and echocardiographic data were available.

All catheterizations were clinical routine examinations that involved an echocardiography within two days and the study included all patients referred to catheterization laboratory without atrial fibrillation and congenital heart disease with echocardiographic data including AT and TR available within the study period. Catheterization was performed by an experienced invasive cardiologist using conventional catheterization technique. A balloon catheter (Becton Dickson Criticath, Franklin Lakes, NJ) was inserted through the right internal jugular vein or right brachial vein. RAP, SPAP, mean pulmonary arterial pressure (MPAP) and the pulmonary capillary wedge pressure (PCW) were measured as well as saturations. Cardiac output (CO) was calculated using thermodilution or Fick^,^s method in which oxygen consumption was calculated. PVR is presented in Wood units (WU) from the following equation: (MPAP-PCW)/ CO. Pressures were registered with a Cathcor system 3.3 (Siemens Elema, Solna, Sweden).

### Ethics

The study was performed with permission from the ethical committee for clinical studies at Uppsala University.

### Statistics

Linear and non-linear regression analyses were performed. Both the 95% confidential intervals for the line and for the population are shown in the figures which also include the r-values. Linear relations with a p-value of < 0.05 were considered significant. Specificity and sensitivity are presented where appropriate. All statistical analyses were performed using Graph Pad Prism software (San Diego, CA). Absolute values are presented as means ± SD.

## Results

### Patient characteristics

In the total group 56% (68 patients) had SPAP above 38 mmHg estimated from echocardiography and the average SPAP was 46 ± 23 mmHg. All patients had a sinus rhythm between 50 and 100 beats/min. None of the included patients had atrial fibrillation, pulmonary stenosis or any other congenital heart disease. In the whole population the average age was 61 years (range 16–89 years) with 67 males and 54 females. For the subgroup of 29 patients with RHC data a more detailed characterization is presented in Table 1. In the subgroup with RHC the average age was 55 years with an equal gender distribution. In this group there was a higher prevalence of PH than in the total study group verified by catheterization (66%) and the majority was in WHO functional class III. During RHC average SPAP was moderately elevated and most patients had no or minor tricuspid or pulmonary regurgitation. The other 92 patients were included directly from routine echocardiography when adequate Doppler curves for repeated measurements of TTVG and AT were available. Patient characteristics and catheterization data are for the RHC subgroup is further described in Table 1.

**Table 1:** Clinical characteristics of the population with right-heart catheterization (n = 29)

**Characteristics**	**n = 29**
Age (years)	55 ± 15
Female/male	13/16
Diagnos of PH	19 (66%)
Diagnos of PAH	11 (38%)
WHO class	
I	0
II	9
III	20
IV	0
SPAP (mmHg)	55 ± 27
MPAP (mmHg)	35 ± 17
PCW (mmHg)	13 ± 7
RAP (mmHg)	8 ± 4
Cardiac output (L/min)	5.2 ± 1.8
Cardiac Index (L/min/m2)	2.7 ± 0.9
PVR (WU)	5.0 ± 4.5
TR	
none-mild	20
moderate	6
severe	3
PR	
none-mild	28
moderate	1
severe	0

Abbrevations: *PH*, pulmonary hypertension; *PAH*, pulmonary arterial hypertension; *WHO*, world health organization; *TR*, tricuspid regurgitation; *PR*, pulmonary regurgitation.

All variables presented as means ± SD unless otherwise stated.

**Figure 1:**
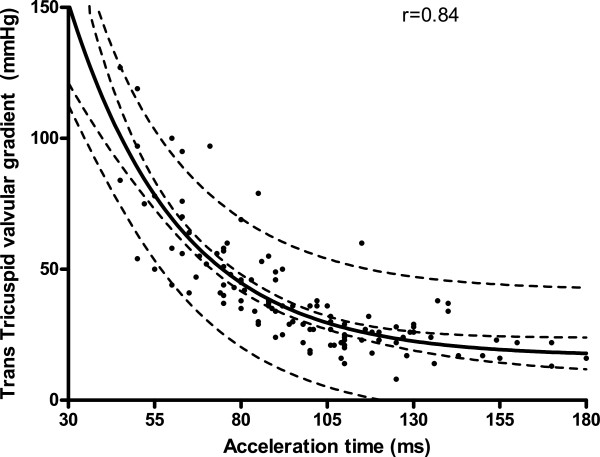
Trans-tricuspid valve gradient (TTVG) in mmHg and acceleration time (AT) in ms with 95% confidence intervals for the line and for the total population shown (n = 121).

**Figure 2:**
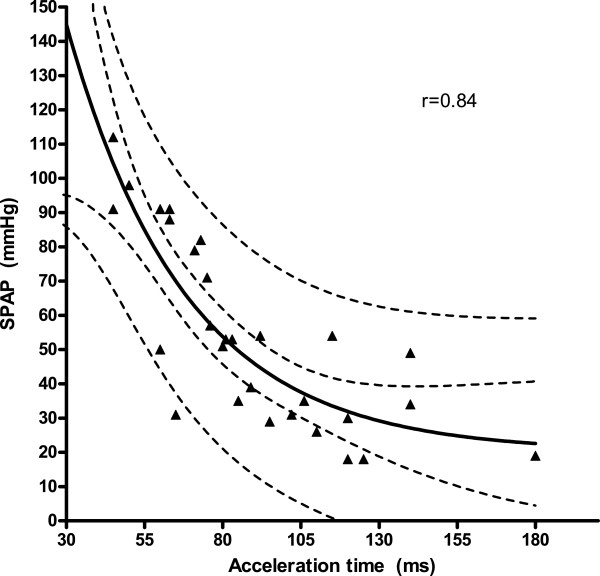
Invasive systolic pulmonary pressure (SPAP) in mmHg and acceleration time (AT) in ms with with 95% confidence intervals for the line and for the catheterization population shown (n = 29).

**Figure 3:**
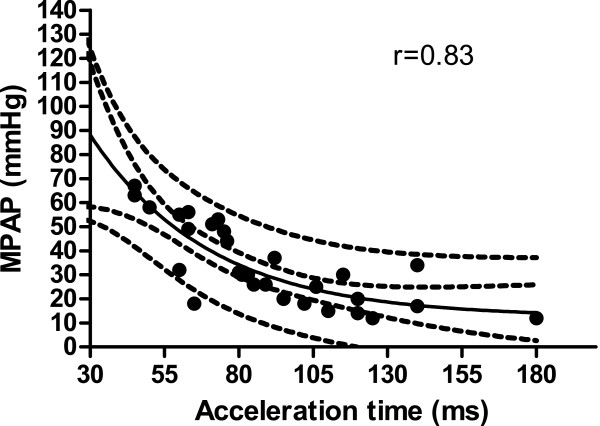
Invasive mean pulmonary arterial pressure (MPAP) in mmHg and acceleration time (AT) in ms with 95% confidence intervals for the line and for the catheterization population shown (n = 29).

The claimed relation of RA pressure and ICV diameter during the breathing cycle could not be confirmed (p = 0.09). Therefore trans-tricuspid valve gradient (TTVG) alone or an estimation of systolic pulmonary pressure (SPAP) by adding 8 mmHg to the TTVG in the RHC-subgroup was used (8 mmHg was the mean RA pressure measured by RHC in the subgroup). In the whole group (n = 121, Figure 1), TTVG and the AT showed a non-linear relation with an r-value of 0.84. The 95% confidence intervals for the line and for the population are shown in Figure 1. The equation for the curve defined as: TTVG = 345.4 · e^-0.0313·AT^ + 16.5 (with AT in ms and TTVG in mmHg). In the subgroup of patients (n = 29) with data from RHC, a similar nonlinear relationship between SPAP and AT was found (r = 0.84, Figure 2). Furthermore, a similar correlation was found between the invasive MPAP and AT from the RHC subgroup (r = 0.83, Figure 3).

**Figure 4:**
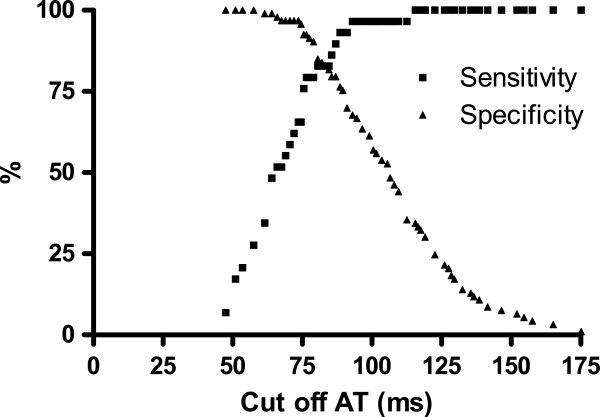
Sensitivity and specificity for different cut off of acceleration time (AT) in ms for detecting pulmonary hypertension with SPAP above 38 mmHg.

The influence of different AT cut-off values on the sensitivity and specificity for detection of either TTVG 30 or 50 mmHg, corresponding to SPAP of 38 or 58 mmHg (Table 2). Using the cut-off for AT of 100 ms to detect SPAP of 38 mmHg (for suspecting PH) resulted in a sensitivity of 89% and specificity of 84%. For SPAP 38 mmHg the sensitivity and specificity are presented for different ATs (Figure 4). As shown, there is an intercept with the lines at AT just below 100 ms, giving both sensitivity and specificity of 88%, whereas a lower cut-off for AT resulted in a lower sensitivity but higher specificity. In the RHC subgroup a higher level than 25 mmHg in invasively measured MPAP, which is corresponding to the SPAP level of 38 mmHg, had a similar sensitivity of 89% and specificity of 80% when using AT below 100 ms as cut-off (not shown in Table). To find a moderately increased TTVG of 50 mmHg or SPAP 58 mmHg in the total study population, similar calculations for different cut-off for AT regarding sensitivity and specificity were performed (Table 2). To detect a SPAP of 58 mmHg, the intercept with the curves occurred at 83 ms with a sensitivity and specificity of 83%.

**Table 2:** Sensitivity and specificity of using different Acceleration times (AT) for detecting systolic Systolic Pulmonary Pressure (SPAP) of above 38 mmHg or above 58 mmHg

**AT (ms)**	**SPAP above 38 mmHg**	**SPAP above 38 mmHg**	**SPAP above 58 mmHg**	**SPAP above 58 mmHg**
	**Sensitivity %**	**Specificity %**	**Sensitivity %**	**Specificity %**
100	89	84	96	57
90	74	98	93	75
75	41	100	75	95

**Figure 5:**
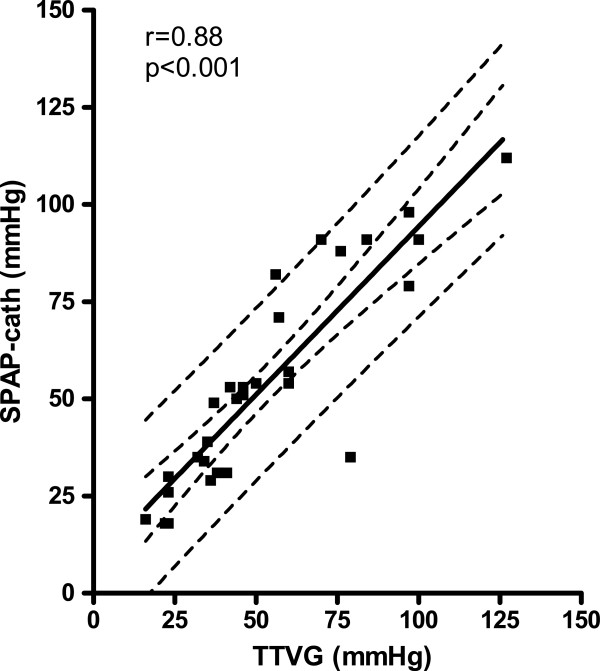
Relation of SPAP from catheterization (SPAP-cath) to trans-tricuspid valve gradient (TTVG) from echocardiography with 95% confidence intervals for the line and for the population shown.

**Figure 6:**
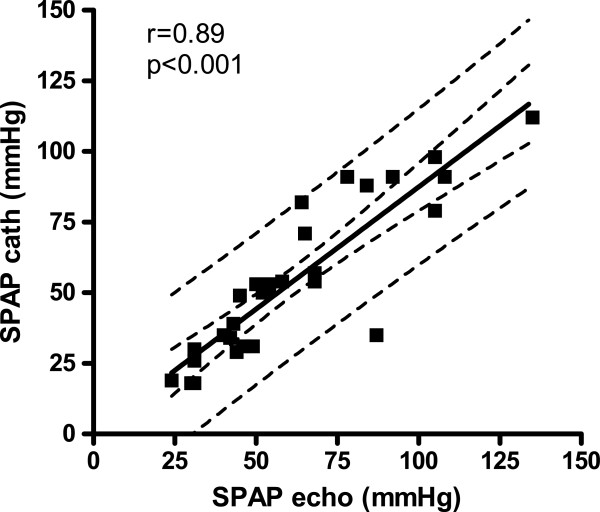
Relation of SPAP from catheterization (SPAP-cath) to estimated SPAP from echocardiography (SPAP echo) with 95% confidence intervals for the line and for the population shown.

The SPAP from RHC correlated significantly with TTVG using a linear regression analysis with r = 0.88 (p < 0.001, n = 29, Figure 5). Corresponding data for estimated SPAP by adding 8 mmHg and SPAP from RHC showed significant linear regression with r value 0.89 (p < 0.001) (Figure 6). The influence of the RA estimation as sometimes used by others [9,11] was not essential for the echocardiographic SPAP correlation with SPAP from RHC, since the correlation with echocardiographic SPAP including RA estimation from ICV collapse in our study was also significant (p < 0.001) with r value 0.87 (not presented in Figure).

**Figure 7:**
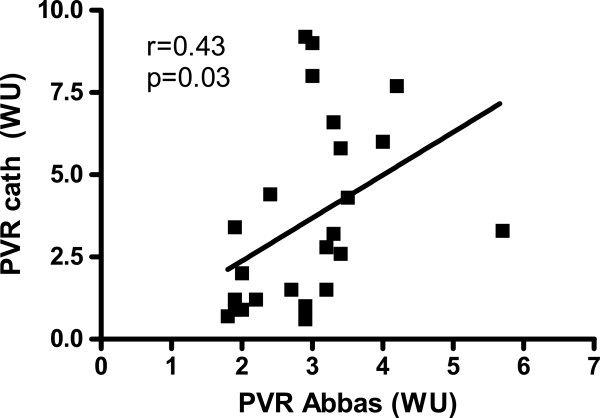
**Relation of pulmonary vascular resistance (PVR) during catheterization in Wood units (WU) to PVR calculated from the equation of Abbas **[7]** (n = 25).**

**Figure 8:**
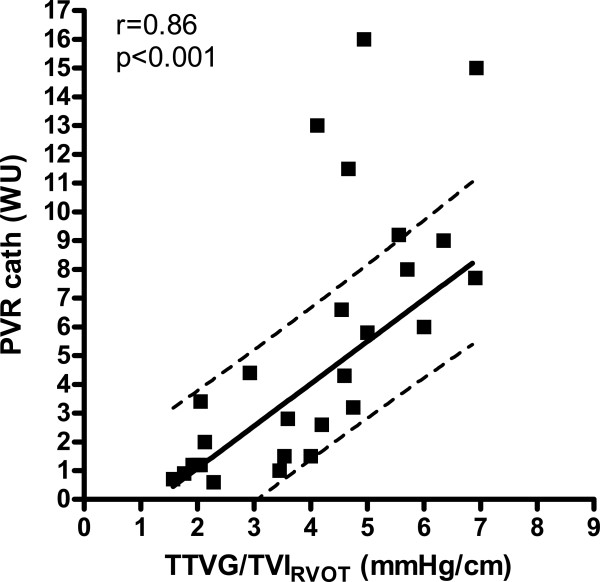
**Relation of PVR during catheterization in Wood units (WU) to TTVG/TVI **_**RVOT **_**(see methods) in mmHg/cm, (n = 25, the four end-stage cases shown but not included in regression analysis) with 95% confidence interval for the population shown.**

In the PVR analysis, four patients were excluded due to a very high PVR (>10 WU) found at RHC representing outliers with low cardiac output regarded as end-stage disease and clearly outside the linear relation between the methods (shown in Figure 7). The PVR calculated from the formula suggested by Abbas [7] did only weakly correlate with PVR derived from RHC (r = 0.43, n = 25, p = 0.03, Figure 8). A stronger linear correlation was found between PVR_RHC_ and the ratio of TTVG and TVI _RVOT_ . Here, the regression showed an r-value of 0.86 (n = 25, p < 0.001, Figure 7). The four outliers clearly are outside the 95% confidence limit for the population and are not included in regression analysis (Figure 7). The equation for this curve was PVR = 1.47 · TTVG/TVI _RVOT_ – 1.86. The linear correlation (r = 0.61) was also significant (p = 0.01, n = 25) but weaker between PVR_RHC_ and the estimated PVR when adding heart rate to the equation (TTVG/TVI _RVOT_ · heart rate) as suggested by Haddad *et al.*[9].

## Discussion

Acceleration time from the Doppler registration in the RVOT close to the pulmonary valve was found to correlate with both the echocardiographic estimation of SPAP and the invasively measured SPAP and MPAP, supported by previous findings [6]. Importantly, the present study evaluated the relationship to invasively measured SPAP, whereas some previous studies have focused on MPAP [11,12]. Moreover, the present study shows that this correlation was equally strong when evaluated in the RHC subgroup with high prevalence of PH. It has previously been shown that AT should be possible to measure in 99% of patients out of which 25% has no measurable TR and thus provide a way of estimating the pulmonary pressure non-invasively [6]. Our data on sensitivity and specificity with different cut-off levels of AT could be used for different clinical situations. An important clinical issue is the screening of high risk populations (SSc and HIV patients for example) with asymptomatic or mildly symptomatic PAH. Previous echocardiographic screening studies using the TR velocity (TTVG) claimed that a diagnosis of PH would be unlikely when TTVG was less than 31 mmHg (TR velocity < 2.8 m/s) and no other echocardiographic signs suggesting PH were present [2,10,13,14]. When a higher cut-off level of TTVG was applied, fewer false positive PH patients were diagnosed on subsequent RHC [1,15] but importantly the number of false negatives was not assessed.

The clear correlation between AT and SPAP (and TTVG) in both the whole population and the RHC subgroup in the present study has important implications for patients without a measurable TR. An AT of less than 100 ms would detect most patients with PH (TTVG >30 mmHg, corresponding SPAP of >38 mmg or MPAP > 25 mmHg) with an acceptable specificity as well. Furthermore, almost all patients with severe PH (SPAP >58 mmHg) could be identified using this cut-off for AT but at the price of lower specificity. However, when screening a high-risk population for PAH, the most important quality would be to have a high sensitivity for selecting patients for further evaluation with RHC. In other clinical scenarios, another cut-off level might be more appropriate. Although some early reports suggested that AT would have a good correlation to MPAP [11,12], the use of AT in clinical practise has remained limited. The study by Yared *et al.*[6] emphasized the availability of AT which together with our data supports the use of AT in clinical and research practise as a promising way of estimating SPAP.

Echocardiographic estimation of SPAP has shown a good correlation to RHC in previous studies [16,17] as well as in our study. However, we suggest that adding RAP estimation to the TTVG did not improve the correlation and used a general 8 mmHg added to the TTVG in agreement with some previous studies when assessing SPAP [6,18,19]. RAP did not correlate sufficiently well with the RHC measured RAP, when using a similar estimation from ICV registrations in our population as shown earlier [9,10]. However, as shown in our study, both ways of estimating SPAP by echocardiography showed a good correlation with the invasively measured SPAP.

Our estimation of PVR using TTVG and TVI _RVOT_ had a better correlation than PVR estimated by the formula of Abbas. Estimating PVR by echocardiography provides additional noninvasive hemodyanamic information. Invasive measurements are essential for exact values for example pretransplantation. Being able to estimate increased PVR noninvasively could have additional value when following patients and changes of treatments. It could be more accurate to use a pressure estimate rather than velocity estimate as in the formula of Abbas when calculating resistance. Different methods could be applied to make the PVR and in our study design it seems more useful to apply the TTVG and TVI _RVOT_ ratio. Regardless of how the PVR estimation is performed the four patients with PVR > 10 WU in the RHC-population were outliers. One reason for this might be that these four patients had poor CO on RHC (CI < 2.0). To detect this noninvasively, the tracing of the pulmonary flow might not be sensitive enough. This agrees with the findings reported by Rajagopalan [8] who showed that PVR estimation using the formula of Abbas [7] was not accurate for PVR levels above 8 WU. Our study also suggests that the PVR estimation should be avoided in patients with PVR > 10 WU or very poor CO.

Despite the clear and interesting findings the retrospective design introduces some limitations. The accuracy when evaluating AT could likely be improved if it was a standard procedure in clinical practise and performed routinely in most patients. Furthermore, even if there was a short time between RHC and echocardiography, the examinations were not simultaneous which might influence data. The number of patients without measurable AT is not available and could reflect some bias. However, it has been shown that the AT measurement is possible to measure in 99% of patients [6] when decided to be routinely performed. The finding that estimated RAP was not significantly correlated to invasive RAP measurement could be due to that they are not simultaneously measured. However, RAP estimated using the ICV did not change the correlation of SPAP to invasive SPAP. Furthermore, our main correlation in the total group involves the TTVG rather than SPAP. Although a prospective study design could improve the quality of measurements, our study with data from regular clinical practise allowed evaluating correlations of clinical importance. On the other hand, when evaluating methods, echocardiography performed in the catheterization laboratory should be done previous to RHC and in a blinded way.

## Conclusions

We suggest that an increased use in clinical practise of AT is justified by its acceptable discriminatory capacity between healthy subjects and patients with pulmonary hypertension. Furthermore, our findings confirm that estimation of SPAP by echocardiography correspond well to the SPAP measured by RHC. Moreover, we found that the PVR from RHC could be estimated by the ratio TTVG/ TVI _RVOT_ .

## Abbreviations

AT: Acceleration time; CO: Cardiac output; CI: Cardiac index; ICV: Inferior vena cava; MPAP: Mean pulmonary arterial pressure; PAH: Pulmonary arterial hypertension; PCW: Pulmonary capillary wedge pressure; PH: Pulmonary hypertension; PR: Pulmonary regurgitation; PVR: Pulmonary vascular resistance; RAP: Right atrial pressure; RHC: Right heart catheterization;RVOT: Right ventricular outflow tract; SPAP: Systolic pulmonary pressure; SSc: Systemic sclerosis; TR: Tricuspid regurgitation; TTVG: Trans-tricuspid valve gradient; TVIRVOT: Time velocity integral of the RVOT; WHO: World Health Organization; WU: Wood units

## Competing interest

We declare that we have no competing interest. GV has received lecture fees from Actelion Pharmaceuticals and Pfizer. SOG has received a consultant fee 2010 from Actelion Pharmaceuticals, however not associated with findings in this study. No support has influenced this study or has had any influence on the study design.

## Authors’ contributions

SOG, MWR and EB planned the study. SOG and MWR made the echocardiographic evaluation and EB and GV the RHC evaluation. MWR and SOG evaluated the statistical analyses and Figure presentations. All took major part in the writing of the article and have read and approved the final form.
